# Tracking Monocytes and Macrophages in Tumors With Live Imaging

**DOI:** 10.3389/fimmu.2019.01201

**Published:** 2019-05-31

**Authors:** Marie Laviron, Christophe Combadière, Alexandre Boissonnas

**Affiliations:** Centre d'Immunologie et des Maladies Infectieuses CIMI, CNRS, Sorbonne Université, Inserm, Paris, France

**Keywords:** tumor-associated macrophages, live imaging, fluorescence reporters, immuno oncology, multiphoton imaging, two-photon microscopy

## Abstract

In most cancers, myeloid cells represent the major component of the immune microenvironment. Deciphering the impact of these cells on tumor growth and in response to various anti-tumor therapies is a key issue. Many studies have elucidated the role of tumor-associated monocytes and tumor-associated macrophages (TAM) in tumor development, angiogenesis, and therapeutic failure. In contrast, tumor dendritic cells (DC) are associated with tumor antigen uptake and T-cell priming. Myeloid subpopulations display differences in ontogeny, state of differentiation and distribution within the neoplastic tissue, making them difficult to study. The development of high-dimensional genomic and cytometric analyses has unveiled the large functional diversity of myeloid cells. Important fundamental insights on the biology of myeloid cells have also been provided by a boom in functional fluorescent imaging techniques, in particular for TAM. These approaches allow the tracking of cell behavior in native physiological environments, incorporating spatio-temporal dimensions in the study of their functional activity. Nevertheless, tracking myeloid cells within the TME remains a challenging process as many markers overlap between monocytes, macrophages, DC, and neutrophils. Therefore, perfect discrimination between myeloid subsets remains impossible to date. Herein we review the specific functions of myeloid cells in tumor development unveiled by image-based tracking, the limits of fluorescent reporters commonly used to accurately track specific myeloid cells, and novel combinations of myeloid-associated fluorescent reporters that better discriminate the relative contributions of these cells to tumor biology according to their origin and tissue localization.

## Introduction

Myeloid cells form a vast and heterogeneous group of cells that play a major role in shaping the tumor microenvironment (TME). Tumor associated macrophages (TAM) represent the most abundant myeloid subset across multiple cancer types, and they generally correlate with poor outcomes. Dendritic cells (DC) in tumors represent a less abundant subset, and contradictory results surround their association with tumor prognosis. DC are classified into subpopulations exhibiting different specificity for priming T-cells (1, 2). Macrophages and DC subsets display a strong overlap of phenotypic markers, adding a high level of complexity to accurately identify them. So far, TAM have been considered to arise primarily from monocyte cells. Recent discoveries regarding tissue macrophage ontogeny challenge this assumption (3). Different tissues of origin are likely to contribute in part to how TAM heterogeneity arises (4–6). Flow cytometry allows qualitative and quantitative characterization of these cells but does not preserve their *in situ* localization to study native cell-cell interactions. In contrast, intra-vital imaging at cellular-scale resolution offers the ability to study cell migration and interactions in living tissue in real-time. It is tempting to consider “truth” what is visible to the eye, and thus direct visualization of cell interactions tends to provide more confidence in the interpretation of a biological process. A main hurdle of this approach rests on the accurate tracking of these cells since the number of available markers are more limited than for flow cytometry and many markers overlap between monocytes, macrophages, DC and even neutrophils, potentially leading to misinterpretations. Moreover, one must keep in mind that imaging experiments usually focus on specific cell subsets, avoiding the potential contribution of the “unseen.” Herein, we review how fluorescent imaging, and more specifically *in situ* live imaging, has contributed to the characterization of TAM and tumor-DC. We discuss limitations of the most common models used for the discrimination and tracking of these different subsets, and we present some perspectives derived from the combination of different fluorescent reporter mouse strains used to unveil microanatomical niches of myeloid subsets in tumors.

## Functional Imaging of Tumor-Associated Myeloid Cells

Microscopy studies represent a necessary approach to truly comprehend the relationship between cells in their physiological environment (7). Beyond the simple identification of cell distribution across the tissue provided by histological analysis, the development of live imaging *in situ* has generated fundamental insights in cellular functions and is termed “functional imaging.” Here, we mention studies based on monocyte and TAM imaging to highlight how this approach has contributed to our knowledge of their function within tumors.

### Functional Imaging of Tumor-Associated Myeloid Cell Dynamics and Interactions With Tumor Cells

Intra-vital imaging of TAM has helped to identify their role in tumor invasiveness and metastasis (8, 9). Direct visualization of fluorescent macrophages and tumor cell lines has revealed CSF1 and EGF-dependent chemotaxis, respectively, (10) and has led to the elaboration of a tumor cell/macrophage cross-talk model (7, 11). *In vitro* imaging is an important complementary approach to study the molecular pathways involved in this model. Beyond paracrine loops, the combination of *in vitro* and *in vivo* imaging has provided evidence that physical contacts between macrophages and tumor cells correlate with invadopodium formation through the induction of RhoA activity on tumor cells (12). The strength of real time imaging is elegantly illustrated by the work of Harney et al showing that the role of Tie2+ perivascular macrophages in this intravasation process is transient and mainly occurs in highly defined microanatomical niches termed “Tumor Microenvironment of Metastasis” (TMEM) (13). Another study has found that macrophages orchestrating early dissemination in breast cancer are CD206^Hi^ and Tie2^+^ and migrate toward tumor cells through CCL2 production by the latter (14).

Macrophages have also been involved in the “streaming cell movement” of tumor cells, defined as the migration of multiple cells in a single file pattern (15). Directional streaming toward the endothelium results from CXCR4 upregulation on TAM and CXCL12 secretion by peripheral fibroblasts (16). Cocultures in 3D-matrices have provided the subcellular resolution to identify a macrophage/tumor cell communication mechanism involving the formation of tunneling nanotubes between the two cell types that is required to induce this directional cell streaming (17). This heterotypic interaction might favor the switch from a mesenchymal migration mode of tumor cells toward an MMP-independent ameboid-like migration as observed in spheroid culture (18). Cytoplasmic exchange between macrophages and tumor cells has been confirmed *in vivo* in zebrafish (19). *In vivo* visualization of migratory activity of TAM, tumor-DC and neutrophils has been observed using differentially ingested dextran particles or differential staining by intravascular injection of fluorescent antibodies in MMTV-PyMT/cfms-EGFP^+^ mice. Sessile cells exhibited strong endocytosis and MMP activity, however TAM and tumor-DC could not be disciminated based on the tested labeling combination (20). Similar labeling approaches have unveiled that migratory capacities of myeloid cells in mammary cancer were less sensitive to hypoxia than regulatory T-cells (21).

Considering macrophage ontogeny and tissue specification has raised the question of their differential function in pathological contexts, particularly in cancer development. Although microglial cells have been considered as the primary TAM subset in brain tumors, it is commonly held that the majority of TAM among many other tumors are monocyte-derived (MoD-TAM) (22). Evidence is recently accumulating that tissue-resident macrophages represent a distinct functional subset from MoD-TAM in other cancer types (16, 23, 24). While resident macrophages were associated with ECM production, recruited macrophages were more involved in the modulation of the adaptive immune response (24), in addition to matrix remodeling and tumor cell clearance following chemotherapeutic treatment (16).

So far, very little information on the role of tissue-resident macrophages in solid tumors is available from imaging studies. The reporter model used in our recent study has been an interesting option for simultaneous tracking of macrophages of different origins in lung tumors (16). MoD-TAM and monocytes tended to accumulate in the periphery of advanced lung tumor nodules and displayed higher displacements than their resident counterparts (16). Their increased migratory behavior also fits with the observation of streaming TAM recruited in a CCR2-dependent manner (25). Accordingly, CCR2-dependent recruited TAM in lung tumors have been associated with remodeling activity and higher tumor cell dissemination (16).

So far, modulating the CCL2/CCR2 axis appears useful in identifying the monocytic origin of TAM. Nevertheless, while the accumulation of tissue resident macrophages has been shown to be CCR2-independent in lung tumors (16), this subset binds CCL2, suggesting that they might respond to a local CCL2 gradient. One should consider that targeting the CCR2 axis may directly or indirectly affect recruited as well as resident TAM. Resident TAM do not necessarly have an embryonic origin but could also arise from local proliferation of MoD-resident macrophages that have progressively colonized the tissue at steady state as observed in several tissues (26). Fate mapping models to track embryonic-derived macrophages by imaging are necessary to determine whether resident TAM are of embryonic origin.

### Functional Imaging of TAM Role in Metastatic Seeding

With the opportunity to track single cells in real time, live imaging has greatly improved our knowledge on the early events of metastatic seeding, in particular through the development of *in vivo* lung imaging (27). Patrolling monocytes have been reported to rapidly engulf tumor material in lung capillaries reducing metastasis development (28). This patrolling activity has also been efficiently monitored using a peritoneal window in colorectal tumors treated with anti-VEGFR2 therapy, highlighting a protumoral activity through neutrophil recruitment (29). Patrolling monocytes do not appear to be the only myeloid cells involved in this process. Rather, a series of sequential waves involving different myeloid subsets are able to uptake tumor material in the lung (30). CCL2-dependent monocyte recruitment has been strongly implicated in metastatic seeding by experiments utilizing CCL2 blockade or global macrophage depletion (31, 32). The relative roles of interstitial lung macrophages and monocyte-derived cells on this early process remain unclear.

### Functional Imaging of TAM and Tumor-DC Interactions With Lymphocytes

Live imaging has also contributed to identifying direct interactions of myeloid cells with T-cells in the TME. Trapping of antigen specific T-cells by myeloid cells in sustained and non-productive interactions has been proposed to favor immunosuppression (33, 34). Macrophage depletion has been associated with increased CD8 T-cell infiltration and improved response to anti-PD-1 “checkpoint” immunotherapy (35). Macrophage/Treg interactions after radiotherapy have also been visualized in a model of head and neck cancer. TNF-mediated cross talk between the two subsets is a proposed mechanism responsible for how an immunosuppressive environment dampens therapeutic efficacy (36). While the vast majority of tumor-infiltrating T-cells seem to be in contact with TAM correlating with poor ability to induce effector functions, Broz et al. have identified a sparse subset of tumor-DC with strong immunostimulatory capacities (2). Recruitment of this subset via NK cell crosstalk mediated by FLT3 ligand and resulting physical interactions defines a positive prognostic factor for anti-PD-1 therapy in melanoma patients (37). Overall, this supports the idea that TAM are usually associated with immune suppressive activity while tumor-DC are more immunostimulatory (38).

Overall, monitoring myeloid cell dynamics, morphology, local distribution in specific TMEM, and interactions with other partners of the TME has unveiled many of their key biological mechanisms. However, the capacity to accurately identify specific myeloid subsets by imaging can be limiting.

## Tracking Myeloid Cells in Tumors

Specific identification of myeloid cells by imaging is challenging because of their heterogeneity, plasticity, and overlapping markers.

*In vivo* antibody injection represents an interesting alternative for cell identification, but there are multiple limitations of this approach. Efficient cell staining is limited by tissue penetration of antibodies, and the persistence of the staining is low due to degradation and recycling activities in living tissues. Finally, the impact of multiple *in vivo* antibody staining on cell dynamics and function cannot be neglected, and findings regarding cell behavior should be interpreted with caution. Fluorescent reporter mice are, thus far, the best option to overcome these limitations. However, the lack of cell-specifc labeling ability still presents a challenge. Promoter-driven fluorescent protein (FP) production is never restricted to a specific subset. Moreover, it is not recommended to associate reporter expression with endogenous protein expression. Therefore, a careful phenotypic characterization of each model using flow cytometry is required to adequately define the imaged cell populations.

Many transgenic mice (listed below) have been developed with various fluorescent reporters to attempt to discriminate specific myeloid populations.

The development of a Csf1r-EGFP transgene (MacGreen) has confirmed that this receptor is expressed in monocytes, tissue-resident macrophages and some populations of DC, such as the Langerhans cells; yet is also present in trophoblasts and granulocytes (39, 40). The deletion of a conserved distal element from the Csf1r promoter on the ΔCSF1R-ECFP reporter (MacBlue) mouse ablated expression in trophoblasts and reduced expression in granulocytes (41). Reporter gene expression is maintained in alveolar macrophages, microglia, and Langerhans cells, however it is ablated in most resident macrophage populations including osteoclasts (42), Kupffer cells (43), and lung interstital macrophages (44). Hawley et al. created a Csf1r-mApple mouse (MacApple) with the same pattern of expression as MacGreen mice (45). Crossing MacApple with MacBlue mice results in specific patterns of fluorescent expression among monocytes and macrophages as observed in the lung and the brain. The authors propose that ECFP expression may be present in cells relying more on IL-34 or CSF2 while ECFP^−^ mApple^+^ macrophages would depend more on CSF1 for their homeostasis (45, 46). The regulation of CSF1R expression requires further investigation.

**Figure 1:**
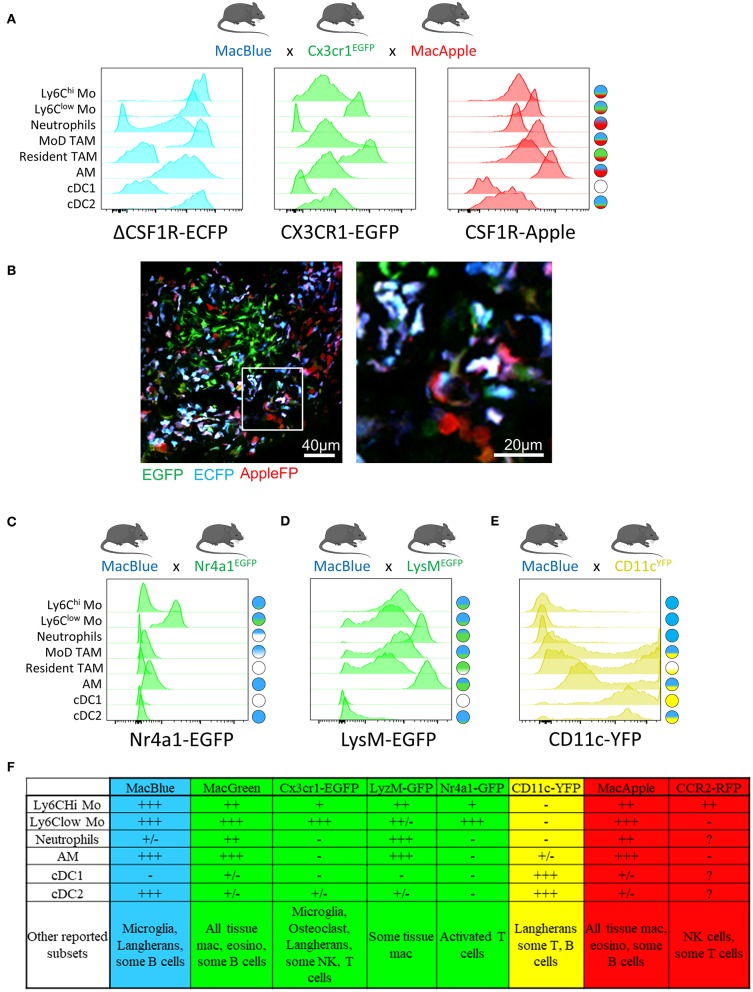
Combination of distinct fluorescent reporter mice identify myeloid cell diversity in the tumor, MacBlue, *Cx3cr1*^*EGFP*^, and MacApple mice were intercrossed to generate a combined fluorescent mouse strain. TC-1 lung carcinoma cell line was inoculated and different myeloid subsets in the lung tumor were analyzed for their relative expression of the fluorescent reporters by flow cytometry **(A)** and tissue distribution by multiphoton microscopy **(B)**. At a single reporter level, the overlap is major between different subsets but the resulting combination of fluorescent reporters for each cell highlights a more specific signature for each subset population (see schematic cell fluorescent signature on the right). **(B)** Left image shows the distribution of distinct myeloid cells in a lung tumor nodule. Right image represents magnification of left image. Discrimination of the distinct subsets is based on the known expression of each reporter seen in **(A)**. Mostly Monocytes and MoD-TAM (Blue/Green/ Red) are distinct from resident TAM (Green), neutrophils (Red) and Alveolar macrophages (Blue/Red). The image was acquired using a Zeiss 7MP multiphoton microscope coupled with a Chameleon Visio II (at 840 nm) and an OPO Mpx (at 1104 nm). **(C)** Combination of Macblue and Nr4a1^GFP^ reporter mice allows the distinction of Ly6Clow from classical monocytes and MoD-TAM. Cell fluorescent signature on the right is generated according to the relative expression of each reporter for all subsets. EGFP is exclusively found in Ly6C^low^ monocytes, ECFP expression is presented in **(A)**. **(D)** Combination of Macblue and LysM^EGFP^ reporter mice allows the distinction of Neutrophils from monocytes and macrophages. EGFP Expression is brighter in neutrophils than in resident interstitial macrophages but similar to alveolar macrophages. Due to spectral overlap between ECFP and EGFP, the accurate discrimination between these subsets can be limited. **(E)** In the *Itgax*^*YFP*^ reporter mice (CD11c^YFP^), the YFP is strongly expressed by classical DC (cDC1) and CD11b+ DC (cDC2) but is also in found in a fraction of Resident and MoD-TAM discriminated by the MacBlue reporter. Therefore, CD11c should not be used as an exclusive marker of DC. For all histogram plots, subsets are defined as: CD11b^+^ Ly6C^hi^ Ly6G^−^ SiglecF^−^ CD64^low^ for Ly6C^hi^ Mo; CD11b^+^ Ly6C^low/−^ Ly6G^−^ SiglecF^−^ CD64^low^ for Ly6C^low^ Mo; CD11b^+^ Ly6G^+^ SiglecF^−^ for Neutrophils; CD11b^+^ Ly6C^−^ CD64^+^ ECFP^+^ EGFP^+^ for MoD TAM; CD11b^+^ Ly6C^−^ CD64^+^ ECFP^−^ EGFP^+^ for Resident TAM; CD11b^+^ Ly6C^−^ CD64^+^ CD11c^+^ SiglecF^+^ for alveolar mac (AM); CD11b^−^ CD11c^+^ CD64^−^ MHC-II^+^ for cDC1; CD11b^+^ CD11c^+^ CD64^−^ MHC-II^+^ for cDC2. **(F)** Table summarizing the relative expression of the different reporters across the indicated immune subsets according to + and-signs. +/– stands for differential expression among one given population.

The *Cx3cr1*^*EGFP*^ reporter mouse (47) is commonly used to monitor patrolling monocytes (29, 48–50) and tissue macrophages (51), but this reporter is also expressed by subsets of NK cells and dendritic cells as well as epidermal T-cells harboring a dendritic-like morphology. EGFP upregulation on subsets of T-cells has been also reported during viral infection (52). Whether tumor-infiltrating T-cells upregulate CX3CR1 must be investigated when using this strain as they can represent an important confounding subset when imaging the TME. We have developed an additional dimension of resolution using the combination of MacBlue x *Cx3cr1*^*EGFP*^ x MacApple reporter mice. This strain provides an improved display of the myeloid compartement heterogeneity in lung tumors, allowing the visualization of recruited, resident interstitial, and alveolar macrophages as well as neutrophils based on differential expression of the fluorescent reporters (Figure 1A). This further highlights microanatomical niches with specific myeloid subset distributions (Figure 1B). Although EGFP expression is lower in classical compared to non-classical monocytes (and has thus been used to track the latter), the discrimination between both subsets by imaging is imprecise. The high expression of ECFP in the MacBlue mouse improves the detection of both subsets, but their discrimination is still not possible (53, 54).

The *Nr4a1*^*gfp*^ fluorescent reporter mouse provides a good marker to monitor non-classical monocytes in the lungs (28). Combination between MacBlue and *Nr4a1*^*gfp*^ might offer an opportunity to simultaneously track both subsets (Figure 1C).

FP expression guided by the *Ccr2* promoter would be expected to preferentially label classical monocytes, but this fluorescent reporter is also highly expressed on NK cells [(55) and personal observation]. NK cells are often abundant in the TME and can lead to misinterpretation of imaging studies using this reporter. Combination with other reporters may therefore improve specificity. For instance, combining *Ccr2*^*RFP*^ and *Cx3cr1*^*EGFP*^ reporters allows tracking of the relative accumulation of CCR2^hi^CX3CR1^low^ and CCR2^low^CX3CR1^hi^ cells in glioblastoma, arguing for distinct origins of TAM in this model (56). As NK subsets also express EGFP in the *Cx3cr1*^*EGFP*^, the risk of NK contamination when imaging and identifyinng myeloid cells in the TME using this mouse strain must be considered.

*LysM*^*EGFP*^ reporter mice display bright expression of GFP based on the lysozyme M locus and are widely used to visualize monocytes and macrophages. However, this marker is also strongly expressed in neutrophils (50, 57). Using this reporter for live imaging is challenging as monocytes, macrophages, and neutrophils are closely related in the TME and the discrimination of these populations requires additional markers. The combination of *LysM*^*EGFP*^ with MacBlue might be considered, but the strong overlap of expression of these two reporters between granulocytes, monocytes and macrophages limits their accurate identification by imaging (Figure 1D).

Mouse strains expressing FP driven by the *Itgax* promoter (CD11c) typically provide very bright fluorescent signal and are available in different colors (58). Although Itgax-based reporters are routinely associated with DC, it is clear that numerous TAM will express the FP and thus prevent the exclusive visualization of DC using this unique reporter (Figure 1E). The combination of *CD11c*^*RFP*^ with *Cx3cr1*^*EGFP*^ in the study by Broz et al has provided an additional dimension to better discriminate DC and TAM in breast tumors (2). The combination of *CD11*^*RFP*^ and *Xcr1*^*venus*^ reporters provides also an alternative to more accurately identify DC by imaging (59).

**Figure 2:**
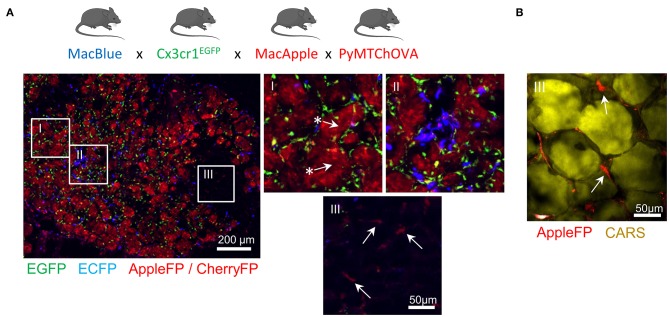
Identification of specific myeloid cell distribution in spontaneous mammary carcinoma. MacBlue x *Cx3cr1*^*EGFP*^ x MacApple mice were crossed with PyMT-ChOVA mice from Engelhardt et al. (33). Briefly this mouse develops spontaneous multifocal mammary tumors expressing CherryFP and Ovalbumin. **(A)** Whole mammary tumors cryo-section shows microanatomical niches of the PyMT tumor with specific enrichment of myeloid cells with distinct fluorescent signatures. EGFP^+^ cells (green) localize at the basal membrane of the mammary carcinomas (region I) and are homogeneously distributed across the neoplasic tissue, ECFP^+^ cells (blue) accumulate in sparse clusters (region II). AppleFP cells cannot be discriminated from CherryFP using these settings but Apple^+^ cells (red) are visualized in the tumor-associated mammary fat pad (region III) confirming the existence of another subset of myeloid cell. Arrows with ^*^ highlight CherryFP^+^ tumor nodules and arrows indicate AppleFP^+^ cells. Images were acquired using a Zeiss epifluorescent microscope (Axio Observer Z1). **(B)** Mammary fat pad-associated AppleFP^+^ myeloid cells were confirmed by CARS imaging (2,846 cm^−1^) allowing the visualization of lipid deposits of adipocytes (in yellow). Image was acquired using a Zeiss 7MP multiphoton microscope coupled with a Chameleon Visio II (at 840 nm) and an OPO Mpx (at 1,104 nm) synchronized by a delay line (Coherent).

Altogether, these transgenic models have demonstrated utility in providing new insights on the dynamics of different myeloid populations (Figure 1F). Furthermore, the combination of different fluorescent reporters appears to be a valid and worthwhile approach to target the cells more accurately. We have already demonstrated that the relative expression of the fluorescent reporter in MacBlue x *Cx3cr1*^*EGFP*^ mice identifies TAM subsets of distinct origins with specific anatomic distribution (16). TAM microanatomical niches are even more marked in the spontaneous mammary tumor model PyMT-ChOVA combined with the MacBlue x *Cx3cr1*^*EGFP*^ x MacApple reporters. Subsets with relative dominant expression of the three FP have been identified (Figure 2A). EGFP^+^ cells are mainly localized to the neoplasic mammary epithelium basal membrane and ECFP^+^ are more clustered in the stroma. In addition to genetic fluorescent reporters, two-photon imaging can be used to generate fluorescence from specific cellular structures without the need of an exogenous fluorescent probe. Coherent anti-Stokes Raman scattering (CARS) imaging, for example, allows imaging of lipid deposits showing that a MacApple^+^ subset is enriched in the adipose tissue of the PyMT tumors (Figure 2B) and favoring the notion of spatial diversity of TAM (60). Whether or not these subsets originate from resident macrophages of the mammary epithelium and surrounding adipose tissue needs further investigation. Second harmonic generation (SHG) is another label-free approach based on the intrinsic optical properties of extracellular structures that has been used to highlight T-cell trafficking in the collagen matrix of the TME (61, 62). Tracking the evolution of collagen density according to tumor stage can be correlated with the functional characterization of TAM, as they are major actors in ECM remodeling. Szulczewski et al. have reported a label-free metabolic imaging protocol allowing for the visualization of NADH and FAD based on their autofluorescent properties. This technique has identified that macrophages express high levels of FAD and are mainly glycolytic, enabling their discrimination from tumor cells without adding any exogenous staining molecule (63). Label-free sensing of biomolecules typically does not result in photobleaching and reflects physiological content and distribution when compared with exogenous fluorescent probes. This label-free imaging also provides an opportunity to obtain information from human samples. As these methods lack specificity, complementary markers are necessary to study myeloid function.

## Concluding Remarks

The delineation of myeloid heterogenity relies on our ability to multiply the number of simultaneously imaged parameters. Although high-dimensional analysis by flow/mass cytometry and single cell transcriptomics is now accessible, accomplishing this characterization with spatiotemporal resolution using optical imaging remains challenging. Because of the strong overlap of commonly used fluorescent reporters between several myeloid subsets, mouse models must be carefully chosen based on the population of interest. The development of spectral unmixing (64) may offer a promising alternative technique to multiply the number of fluorescent parameters recorded simultaneously, but so far has been restricted to analysis of fixed tissue. The use of imaging windows allows longer-term tracking of cellular behavior (65). This approach may also contribute to better understand myeloid functions over time and in response to therapy. Tracking myeloid cell subsets using combinations of complementary approaches, such as *in vivo* fluorescent antibody labeling, dextran uptake, endogenous fluorescent reporters, and label-free optical imaging processes, is likely to yield a full appreciation of the phenotypic and functional diversity of TAM and DC. Fate mapping models to label embryonically derived macrophages might additionally identify tumor myeloid cell origin and will certainly be the goal of imaging studies in the near future. Despite some complexity that can dampen the accurate identification of myeloid subsets in the TME, previous studies have been extraordinarily rewarding in our understanding of tumor-associated myeloid cell biology.

## Data Availability

The datasets generated for this study are available on request to the corresponding author.

## Ethics Statement

Mouse experimentation were approved by the French animal experimentation and ethics committee and validated by “Service Protection et Santé Animales, Environnement” with the number A-75-2065.

## Author Contributions

ML performed the experiments. All authors wrote the manuscript.

### Conflict of Interest Statement

The authors declare that the research was conducted in the absence of any commercial or financial relationships that could be construed as a potential conflict of interest.
